# Water exchange across the blood–CSF barrier: A systematic review

**DOI:** 10.1177/0271678X251413926

**Published:** 2026-02-07

**Authors:** Trine Hjørnevik, Per Kristian Eide

**Affiliations:** 1 Department of Physics and Computational Radiology, Oslo University Hospital, Oslo, Norway; 2 Faculty of Medicine, Institute of Clinical Medicine, University of Oslo, Oslo, Norway; 3 K.G. Jebsen Centre for Brain Fluid Research, University of Oslo, Oslo, Norway; 4 Department of Neurosurgery, Oslo University Hospital—Rikshospitalet, Oslo, Norway

**Keywords:** Cerebrospinal fluid, CSF flow, blood–CSF–barrier, water, solvent, solutes, diffusion

## Abstract

Interest in cerebrospinal fluid (CSF) physiology and its relevance to neurological disease has increased markedly in recent years. Classical descriptions portray CSF as a unidirectional flow from the choroid plexus to the dural venous sinuses and rarely distinguish between its solutes and the solvent (water) component, which constitutes ~99% of CSF. We conducted a systematic literature review to evaluate current evidence for water exchange between CSF and blood across the blood–CSF barriers (BCSFB). Eighteen studies met the inclusion criteria: 15 in experimental animals and six with humans, spanning more than 70 years and employing diverse methodologies. This literature review shows that CSF water moves freely and bidirectionally between CSF and blood across multiple BCSFB sites along the craniospinal axis, including the choroid plexus, ependymal surfaces, pial vessels, and perivascular spaces. The net direction of movement varies locally with hydrostatic, osmotic, and molecular gradients that transiently favor either inflow or outflow. Blood–CSF water exchange occurs predominantly by diffusion and is modulated by aquaporins and local vascular forces. These findings challenge the classical concept of unidirectional CSF production and absorption, supporting instead a dynamic equilibrium where distributed, gradient-driven water flux maintains brain water homeostasis.

## Introduction

The year 2025 marks several milestones in cerebrospinal fluid (CSF) research. In 1825, François Magendie published *Mémoire sur un liquide qui se trouve dans le* crâne, introducing the concept of CSF,^1^ and a century later, in 1925, Harvey Cushing coined the term “*third circulation*.”^2^ Over time, CSF has been regarded as a single fluid with well-defined production sites (primarily the choroid plexus), fixed circulatory pathways (from the ventricles to extracerebral exit routes), and dedicated absorption sites (mainly the dural venous sinuses).

This classical textbook model has been increasingly questioned.^3,4^ Recent work highlights discrepancies between the traditional “third circulation” and emerging views of CSF as a fluid undergoing exchange at multiple sites.^4^ In parallel, the discoveries of the glymphatic and meningeal lymphatic systems have emphasized the importance of CSF in solute transport, metabolic waste clearance, and immune surveillance.^5^ Although these discoveries have shifted our understanding of CSF physiology, the prevailing framework still largely treats CSF as a uniform solution produced primarily by the choroid plexus.^6,7^

CSF consists of ~99% water (H_2_O) and ~1% solutes, including electrolytes, trophic factors, and metabolic by-products. Yet the water component is seldom considered separately in physiological models, and many techniques for measuring CSF “production” implicitly treat CSF as a homogenous entity. A recent methodological review underscored this limitation.^8^

According to the Starling principle, water and solute exchange across systemic capillaries are closely coupled.^9,10^ Whether this paradigm fully applies to the vasculature of the central nervous system (CNS) remains uncertain; it is unclear to what extent water may cross CNS barriers independently of solutes. A clearer distinction between CSF solvent and solute components is therefore essential, especially regarding water exchange between CSF and blood across the blood–CSF barriers (BCSFB). To address this gap, we conducted a systematic literature review to evaluate current evidence for water exchange between blood and CSF in terms of (i) direction (production vs absorption), (ii) magnitude, (iii) anatomical distribution along the craniospinal axis, and (iv) regulatory mechanisms (driving forces, water-solute dissociation, and molecular pathways).

In this review, we distinguish between human and animal data and examined the influence of neurological disease on these processes. Furthermore, given the current interest in the glymphatic system and the central role of aquaporine-4 (AQP4) in glymphatic flux, we also consider how the identified evidence aligns with or challenges the glymphatic framework.

## Materials and methods

### Search strategy and selection criteria

Firstly, we conducted a comprehensive literature search across EMBASE (Ovid), MEDLINE (Ovid), and Scopus (Elsevier), with no restrictions on language, publication date, document type, or publication status. This search was guided by the following research question: *What is the current evidence regarding water exchange between CSF and blood in terms of (i) direction (production vs absorption), (ii) magnitude, (iii) anatomical distribution along the craniospinal axis, and (iv) regulatory mechanisms (driving forces, water-solute dissociation, and molecular pathways)?* Secondly, a citation search based on the initial literature search result was conducted using Citation Chaser, Research Rabbit, Scopus, and Web of Science.

The final search was completed in April 2025, and the complete search strategy is summarized in Supplementary Table 1.

Both authors independently screened titles, abstracts, and full texts using Covidence systematic review software (Veritas Health Innovation, Melbourne, Australia), including only original research articles published in English. The full-text exclusion criteria were as follows: (1) full text not available after reasonable effort; (2) not a primary research study, including book chapters; (3) outcomes not relevant to the review question; and (4) not relevant to the review question. Any discrepancies were resolved between the two reviewers.

### Data extraction

Extracted data included: (1) species studied, (2) disease model or diagnosis, (3) methods and outcome measures used to assess blood–CSF water exchange, (4) direction of water movement (CSF → blood or blood → CSF), (5) magnitude measures of blood–CSF water exchange, (6) anatomical site of exchange (e.g. brain ventricles, spinal cord), (7) mechanisms regulating the water exchange, and (8) author’s interpretation of findings. Results were analyzed separately for animal and human studies.

## Results

### Study selection

**Figure 1.**
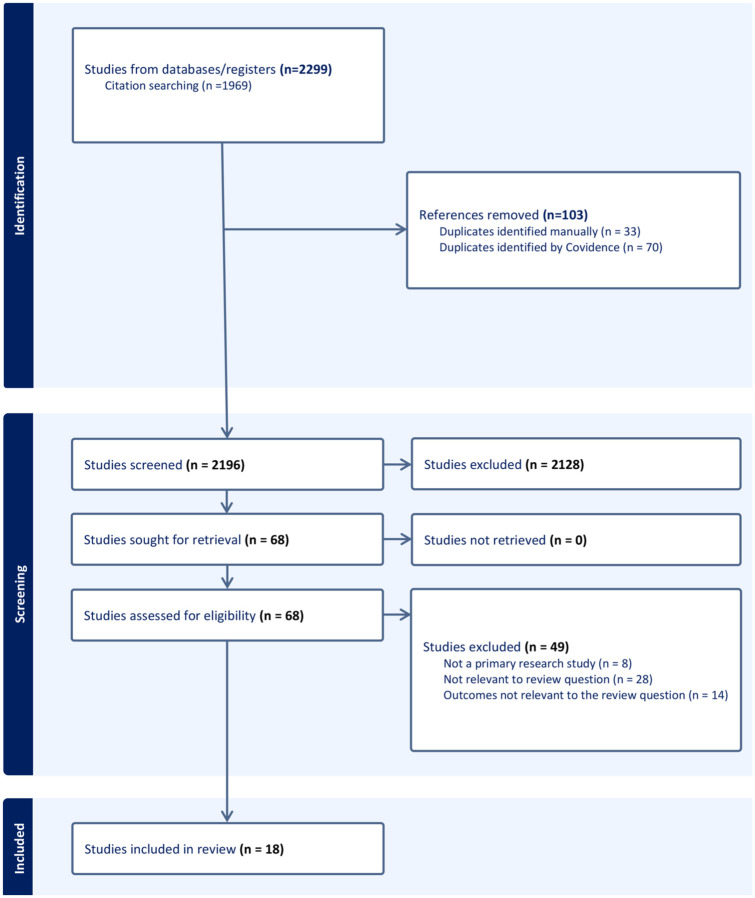
PRISMA 2020 flow diagram for the systematic review that include searches of databases. The PRISMA plot^11^ with included and excluded studies.

A total of 2,299 articles were identified through the systematic literature and citation search, and imported into Covidence for screening. After automatic and manual removal of 103 duplicates, 2,196 titles and abstracts were screened for relevance. Sixty-eight full-text manuscripts were assessed for eligibility, of which 18 met all inclusion criteria and form the evidence base for this review. Among these, 15 studies reported animal data, six included human data, and three contained both. The full list of included studies is provided in Supplementary Table 2. Figure 1 displays the PRISMA 2020 flow diagram for the review (http://www.prisma-statement.org/PRISMAStatement/PRISMAStatement.aspx).^11^

The included studies span more than 70 years of research. Table 1 summarizes the 15 animal studies and Table 2 summarizes the six human studies investigating blood–CSF water exchange. Over this period, the tracers and imaging methods evolved substantially: heavy water (D_2_O) in the 1950s,^12–14^ tritiated water (T_2_O) from the 1960s to the early 2000s,^15–21^ and H_2_^17^O magnetic resonance imaging (MRI) techniques during the past decade.^22–25^ More recently, arterial spin labeling (ASL) MRI has been applied to investigate water transfer from blood to CSF.^26–29^

**Table 1.** Animal studies on water exchange between blood and CSF.

Reference	Methodology	Main results	Author interpretation
12	*Species:* Dogs (*n* = NR). *Intervention:* Heavy water (D_2_O, 1 mL/kg) injected IV and analyzed with densitometry or mass spectrographic method from CSF samples from CV, CM, and lumbar SAS, and tissue from brain, cerebellum and spinal cord. *Outcome measures:* Estimation of half-time for any compartment to reach blood D_2_O concentrations, i.e., half-time of exchange from blood to tissue or CSF	Half-time for passage of D_2_O from blood to tissue: Cerebral gray matter: 12 s. Cerebral WM: 20 s. Cerebellum: 12 s. Spinal cord: 25 s. Ventricular CSF 8 min. Cisternal CSF: 3 min. Lumbar CSF 7 min	All the brain water, intracellular and extracellular, is in a free and extraordinarily rapid equilibrium with the water of the blood. The CSF water-component is continually exchanging at all surfaces of the brain and spinal cord by free diffusion. This exchange is not necessarily to be identified with the formation of CSF as it involves only the water-component of CSF. Exchange between CSF and blood relates to the surface are-volume ratio
13	*Species:* Monkeys (*n* = NR). *Intervention:* D_2_O (up to 1.9 mL/kg) injected into CM and analyzed using densitometry or mass spectrographic method from blood. *Outcome measures:* Estimation of half-time for D_2_O to reach blood, i.e., half-time of exchange from CSF to blood	Quantitative values NR (safety study). Intracisternal D_2_O in a volume of 1.9 mL/kg had no adverse effects, appeared in the blood at high levels and rapidly fell to an equilibrium level	There is rapid exchange of water between CSF and blood
14	*Species:* Dogs (*n* = NR). *Intervention:* Different tracers (D_2_O, ^24^Na, ^42^K, and ^131^I-albumin) injected IV and analyzed using densitometry or mass spectrographic method from ventricular CSF. *Outcome measures:* Appearance of tracer in ventricular CSF before and after removal of CP. Specific activity (normalized to activity in serum at 3 h post injection)	The exchange of each tracer between blood and CSF occurs at different rates, and likely independently, water fast and larger molecules slowly. Comparable results between dogs and humans	Equilibrium of D_2_O after about 100 min, while the other tracers showed different profiles CP not important for water/ionic/molecular exchange as CP removal did not affect D_2_O, ^24^Na, or ^131^I-albumin exchange None of these tracers represent CSF as a whole. Each tracer must be considered by itself and accumulation of CSF treated as a separate phenomenon. CSF accumulation should be considered separate from tracer ion exchange
15	*Species:* Goat (*n* = NR). *Intervention:* Tritiated water (T_2_O; 1.5–2 mL/min, 70–120 mL) injected into CV (ventriculo-cisternal perfusion) and samples from ventricular CSF analyzed using liquid scintillation counter. *Outcome measures:* Assessment of inflow-outflow over time	Goat 1: Inflow 3500 ± 30 count/min/mL. Outflow 1590 ± 30–1810 ± 30 counts/min/mL (depending on CSF pressure −8 to 28 cm H_2_O) Goat 2: Inflow 4130 ± 30 count/min/mL. Outflow 1720 ± 50–1840 ± 50 counts/min/mL (depending on CSF pressure −10 to 26 cm H_2_O) Permeability for T_2_O at ventricular wall in goat (*n* = 3): Permeability coefficient 1.7 ± 0.5 cm^3^/min. In comparison: ^24^Na (goat, *n* = 4): 0.18 ± 0.01 cm^3^/min. ^42^K (goat, *n* = 5): 0.80 ± 0.13 cm^3^/min Permeability for T_2_O at ependymal lining of ventricular system: 28 × 10^5^ cm/s	Diffusion accounts for most of T_2_O lost from ventricular CSF, and is highly related to pressure, i.e., ventricular efflux of water increases with increasing hydrostatic pressure
17	*Species:* Monkeys (*n* = 4). *Intervention:* T_2_O (0.1 µCi/lb BW), ^22^Na, and ^131^I-albumin injected into CM and analyzed in blood from cortical vein, SSS, and brachial artery using liquid scintillation counter. *Outcome measures:* Assessment of passage from CSF to blood and the establishment of equilibrium (% injected dose/0.5 mL blood)	No quantitative measures of CSF to blood exchange given. Extremely rapid passage of T_2_O from CM CSF to blood. Within 10 min similar levels of T_2_O were found in sagittal sinus, vein of Labbe and arterial blood (after 5 min higher activity in the arterial blood than sagittal sinus). This was different for ^22^Na that obtained equilibrium after 45 min. ^131^I-albumin was even slower and seemed to be absorbed to the SSS	T_2_O passes nearly immediately from CSF of CM to blood. Na^+^ shows a slower exchange to blood via many regions, while larger substances are absorbed in the parasagittal regions
18	*Species:* Dogs (*n* = 9). *Intervention:* T_2_O (12–25 µCi/mL) and ^22^Na injected into CSF of a compartmentalized SAS (small CSF-filled cylinder within SAS) and analyzed in blood from SSS using liquid scintillation spectrometer. *Outcome measures:* Radioactivity of tracer (half-time; *T*_1/2_) in blood examined before and after removal of pial vessels	The average half-time (*T*_1/2_) for appearance of T_2_O in venous blood was 14 s (range: 5–28 s) The rate of T_2_O appearance in the SSS blood is fast during the first 30–50 s suggestive of water passage from the pial vessels to the blood. In the absence of pial vessels, no appearance of T_2_O in the venous blood until the 60 s sample (since T_2_O had to pass via brain tissue to reach blood) No appearance of ^22^Na in blood	Pial vessels transport water, but not Na^+^, from CSF to blood. Pial vessels may serve as a clearance function The authors conclude that the secretory function of CP is not necessary for the formation of CSF and propose it might be an active clearance site for substances from CSF; the production and regulation of the fluid composition being reserved to endothelial cells of all the cerebral vasculature: pial vessels, parenchymal capillaries and choroidal capillaries
19	*Species:* Cats (*n* = 39). *Intervention:* T_2_O (1.67 µCi/mL) injected via ventriculo-cisternal perfusion and radioactivity measured ex vivo in brain tissue using liquid scintillation counter. Effect of arginine vasopressin (AVP). *Outcome measures:* T_2_O in tissue as a measure of subependymal capillary permeability	The ratio of tissue to plasma T_2_O concentration was 0.011 ± 0.001 in both controls and animals treated for 2 h with AVP No difference in mean water content was observed for control and AVP-treated animals (78.9 ± 0.5 vs 80.8% ± 0.4% (mean ± SE)) Ependymal permeability was estimated in control animals to be 2.3 ± 0.9 × 10^−4^ cm/s AVP hormone treatment caused increased distribution space of T_2_O, increased capillary transfer half-time, and increased T_2_O clearance slope Distribution space (%) of T_2_O: (i) Controls: 28.3% ± 6.2% and (ii) AVP group: 52.0% ± 6.6% Capillary transfer half-time of T_2_O: (i) Controls: 1.76 ± 0.23 min and (ii) AVP group: 1.13 ± 0.09 min Ependymal permeability of T_2_O: (i) Controls: 2.3 ± 0.9 × 10^−4^ cm/s and (ii) AVP group: 9.7 ± 4.0 × 10^4^ cm/s	The ependymal surface forming the interface between CSF and ISF limited movement of T_2_O from CSF to blood The authors discuss that water diffusion is restricted at fluid-tissue interfaces in the brain and that the capillary surface area in a volume of tissue close to the ventricle is greater than that of the apical surface of the ependymal cell, which suggest that the ependymal complex is a rate-limiting step in the transfer of water from CSF to blood AVP increased ependymal permeability to T_2_O, increased distribution space, and reduced capillary transfer half-time. Vasopressin increases water absorption capacity
16	*Species:* Cats (*n* = NR). *Intervention:* T_2_O and ^3^H-inulin injected into CV or cortical SAS and radioactivity measured in vein, artery, and CSF using liquid scintillation counter. Three experimental setups: (i) *Acute:* T_2_O (0.125 mCi/mL, 1.77 µL/min) to lateral CV or cortical SAS over 3 h with fluid sampling from sinus confluence, arterial blood, and CM. (ii) *Sub-chronic:* T_2_O (0.50 mCi/mL, 1.0 µL/h) to lateral CV over 5 days in freely moving cats. Sampling from CSF of cortex, CM, thoracic and lumbar CSF, and from sinus confluence and arterial blood. (iii) IV T_2_O (0.017 mCi/mL) and sampling from CM and arterial blood. *Outcome measures:* Radioactivity in fluid samples to assess passage of CSF in ventricles and SAS to periventricular capillaries and to internal cerebral veins/vein of Galen. Radioactivity in CM CSF from blood. Counts per minute/50 µL	(i) *Acute experiments:* Higher concentration of T_2_O in confluence plasma water than in cisternal CSF and arterial plasma water (the latter compartments are equal) After infusion into cortical CSF, T_2_O concentration in confluence plasma was several times higher than in arterial plasma and cisternal CSF (ii) *Sub chronic experiments:* No statistical difference in T_2_O concentration was detected between various CSF compartments (cisternal, cortical, thoracic, and lumbar CSF), confluence and arterial plasma, and urine. (iii) *IV injection:* Equilibrium between CM and arterial plasma in about 5 min ^3^H-inulin exhibited a different flow pattern with bidirectional transport and slow removal	Absorption of water in brain ventricles is much higher than its postulated unidirectional flow between lateral ventricle and CM. Ependymal cells/lining provides no strong barrier to transport of water from ventricular CSF to periventricular capillaries T_2_O passes from SAS across pia mater and is absorbed into cerebral microvessels, which drain to adjacent SSS, and by blood flow it reaches the confluence of sinuses Water is formed and absorbed locally, no unidirectional movement High concentration of T_2_O in the vein of Galen compared to lower concentration in arteries and cisternal CSF suggest direct transport from subependymal arteries to veins/vein of Galen
			The authors conclude that experiments with T_2_O support the idea that CSF volume (water) does not flow unidirectionally along CSF spaces but is locally absorbed in adjacent microvessels located in brain parenchyma Neither the net formation of CSF volume (water) in brain ventricles nor its unidirectional flow in SAS are present. Thus, it appears that the formation and absorption of CSF in ventricles are in balance
22	*Species:* Mice (SP bearing transgenic AD model (APP-PS1) mice (*n* = 5) and wild type (WT) mice (*n* = 5). *Intervention:* In vivo tracing of water flux using JJVCPE MRI and IV H_2_^17^O (20% in 0.2 mL saline). *Outcome measures:* Influx of H_2_^17^O to cerebral cortex, basal ganglia and third ventricle, measured as average % intensities at steady state, as compared to baseline	The concentration of H_2_^17^O in brain plateaued after 20 min In WT mice, penetration and subsequent steady concentration of H_2_^17^O in CSF of the third ventricle is significantly higher than compared to brain parenchyma (cortex and basal ganglia) Water (H_2_^17^O) flux to CSF of third ventricle is reduced in AD mice as compared to WT mice No quantitative measures of blood to CSF exchange given	A significant reduction of H_2_^17^O influx to CSF was observed in AD mice It is proposed that ventricular CSF plays a role as lymph nodes in neutralizing toxicity of some proteins, and that dysfunction in the AQP4-related “lymphatic” system of the brain is significantly correlated with formation of SPs
24	*Species:* Mice (AQP1-KO (*n* = 6) and AQP4-KO (*n* = 6) and WT (*n* = 6) mice). *Intervention:* In vivo tracing of water flux using JJVCPE MRI and IV H_2_^17^O (20% in 0.2 mL saline). *Outcome measures:* Influx of H_2_^17^O to cerebral cortex, basal ganglia and third ventricle, measured as average % intensities at steady state, as compared to baseline	The concentration of IV H_2_^17^O in brain plateaued after 20 min In WT mice, penetration and steady state concentration of H_2_^17^O was significantly higher in CSF of third ventricle than brain (cortex and basal ganglia) The behavior of water (H_2_^17^O) molecules was virtually identical in WT and AQP1-KO mice Penetration and steady state concentration of H_2_^17^O in CSF of the third ventricle was significantly reduced in AQP4-KO mice No quantitative measures of blood to CSF exchange given	It is proposed that AQP4, not AQP1, is crucial for water production to ventricular CSF Water movement within the pericapillary spaces, rather than CP and arachnoid villi, is essential for CSF volume homeostasis Water movement from the general circulation into the brain has two different routes, one of which is critical for CSF homeostasis and dependent on the presence of AQP-4
25	*Species:* Mice (WT mice, *n* = 10). *Intervention:* In vivo tracing of water flux using JJVCPE MRI and IV H_2_^17^O (40% in 0.2 mL saline), with (*n* = 5) or without (*n* = 5) the AQP4 facilitator TGN-073. *Outcome measures:* Influx of H_2_^17^O to cerebral cortex, basal ganglia, and third ventricle measured as average % intensities at steady state, as compared to baseline	The concentration of IV H_2_^17^O in brain plateaued after 20 min Water flux to cortex (but not CSF or basal ganglia) was facilitated by the AQP4 facilitator TGN-073, resulting in reduced concentration of H_2_^17^O in cortex due to higher turnover of interstitial fluid in cerebral cortex associated with AQP4 facilitation. Facilitation of AQP4 increased the turnover of water from cortical into pericapillary spaces No quantitative measures of blood to CSF exchange given	It is suggested that facilitation of AQP4 functionality is a promising pharmacological target in the prevention and treatment of AD
23	*Species:* Mice (AQP4-KO (*n* = 8) and WT (*n* = 6) mice). *Intervention:* In vivo tracing of water flux using ^17^O-MRI with IV H_2_^17^O (150 µL 0.9% saline with 12.6% enriched ^17^O). *Outcome measures:* Flux of H_2_^17^O from blood to CSF, measured as change in ^17^O signal within lateral ventricles. *Measures:* Peak and time of peak. Steady state. Time constant for washout	Peak increase in ^17^O signal in brain and CSF within 30 s in both AQP4-KO and WT mice In CSF, the time constant was 2.82 ± 0.48 and 2.94 ± 0.76 min for WT and AQP4-KO mice, respectively (non-significant) The peak ^17^O signal was 17% lower (*p* < 0.05) in brain of AQP4-KO mice suggesting reduced H_2_^17^O uptake The steady state ^17^O signal showed 20% reduction in brain of AQP4-KO mice About 22% increase in capillary density in AQP4-KO mice	Reduced AQP4-mediated water movement across BBB accounts for decreased H_2_^17^O uptake and retention in AQP4-KO mice The AQP4 water channel is involved in oxygenation of tissue
26	*Species:* Rats (*n* = 12). *Intervention:* CASL MRI. *Outcome measures:* BCSFB water flow (mL/min/100 g) providing a measure of blood-to-CSF water flow in CP	The BCSFB water flow was only 25% of the CP tissue perfusion values The duration for the arterial blood to arrive at the CSF through the BCSFB water flow was nearly doubled compared to that of the cortical perfusion The BCSFB mediated delivery of arterial blood water from the CP into ventricular CSF is directly proportional to the CP tissue perfusion, however, the rate of exchange is not one to one BCSFB water flow depends on the anesthetic used. BCSFB water flow: 65 ± 17 (DEX-I) and 76 ± 15 (ISO) mL/min/100 g BCSFB water flow arterial transit time (ms): 1083 ± 366 (DEX-I) and 746 ± 463 (ISO)	Proof-of-concept study where the results support the BCSFB water flow as a surrogate marker for CSF secretory function Whether or not the origin of BCSFB water flow reflects CSF secretory function only, driven by active water transport, or represents a combination of CSF secretion and diffusive water exchange remains an open question
27	*Species:* Rats (SHR model (*n* = 6) and controls (*n* = 6)). *Intervention:* BCSFB ASL MRI. *Outcome measures:* BCSFB water flow (mL/min/100 g) providing a measure of blood-to-CSF water flow in CP and lateral ventricles	BCSFB-mediated water delivery rate to ventricular CSF was reduced in SHRs compared to healthy rats (14.4 ± 1.92 vs 9.22 ± 1.20 µL/min (35.8% reduction)) Altered T1 CSF suggest altered CSF composition in arterial hypertension	A proof-of-concept study of a new method for estimation of BCSFB-mediated water delivery rate to ventricular CSF Spontaneous hypertension is accompanied with reduced BCSFB-mediated labeled arterial water delivery to ventricular CSF The observed functional impairment at the BCSFB suggests that the CP-BCSFB-CSF system may represent a key site of brain vulnerability to systemic hypertension
29	*Species:* Mice (normal controls (*n* = 31) and 3XTg AD-model (*n* = 40) at different ages). *Intervention:* ASL–MRI. *Outcome measures:* Total BCSFB mediated water delivery at CP (and lateral ventricles)	The BCSFB-mediated total water delivery was significantly higher in AD-model mice compared to controls at all ages: 8 weeks (pre-clinical): Controls: 0.55 ± 0.09 µL/min. AD: 1.13 ± 0.10 µL/min 14 weeks (sub-clinical): Controls: 0.80 ± 0.07 µL/min. AD: 1.34 ± 0.04 µL/min 20 weeks (early-clinical): Controls: 0.81 ± 0.06 µL/min. AD: 1.39 ± 0.09 µL/min 32 weeks (mid-clinical): Controls: 0.74 ± 0.11 µL/min. AD: 1.32 ± 0.09 µL/min	Proof of concept CP function is altered in the early and mid-stages of AD, i.e., CP may play an important role in the early pathophysiology of AD due to (1) breakdown of BCSFB integrity and (2) an increase in CP perfusion for boosting clearance due to the presence of adherent proteins

AQP: aquaporin; ASL: arterial spin labeling; BCSFB: blood–CSF-barrier; CASL: continuous arterial spin labeling; CM: cisterna magna; CP: choroid plexus; CSF: cerebrospinal fluid; CV: cerebral ventricles; DEX-I, dexmedetomidine; HC: hydrocephalus; ICV: intracerebroventricular; ISO, isoflurane; IV: intravenous; JJVCPE, JJ vicinal coupling proton exchange; MRI: magnetic resonance imaging; NR: not reported; SAS: subarachnoid space; SHR: spontaneous hypertension rat; SP, senile plaque; SSS: superior sagittal sinus; TBW: total body water.

**Table 2.** Human studies on water exchange between blood and CSF.

Reference	Methodology	Main results	Author interpretation
12	*Subjects:* (i) “Normal” subjects (8 months–87 years, *n* = 5) who had a unilateral subdural hematoma removed 3–6 weeks before. (ii) Patients with radical bilateral CP removal (*n* = 2). (iii) Patients with isolated spinal SAS (*n* = 2). (iv) Infants with HC (*n* = 12). *Intervention:* Heavy water (D_2_O; 1 mL/kg) injected IV and analyzed densitometry or mass spectrographic method from blood of artery and SSS, CSF from CV, CM, and spinal SAS, and tissue from brain, cerebellum and spinal cord. *Outcome measures:* Estimation of half-time for any compartment to reach blood D_2_O concentrations, i.e., half-time of exchange from blood to CSF	(i) “Normal” subjects: Speed of appearance of D_2_O in CSF: CM > CV > lumbar SAS. After equilibrium of D_2_O in blood and CSF, rate of disappearance at same rate from both CSF and blood Half-times: CV (2–37 min), CM (1.5–6 min), lumbar SAS (7–38 min) (ii) CP removal had no impact on water exchange (iii) Lumbar half-times in subjects with isolated spinal SAS: Lumbar SAS (33 and 20 min) (iv) Half-times in infants with hydrocephalus: CV (60–245 min), lumbar SAS (9–36 min) Half-times increase with age	The CP cannot be the sole site for the entry of water into CSF given the findings of the early appearance of D_2_O in CM, the lack of change in D_2_O exchange after removal of the CP, and the independence of water exchange at lumbar SAS from its connection with the SAS at higher levels The appearance of D_2_O in CSF is governed by the ratio between surface area and volume of any compartment Circulation of CSF through an intact SAS pathway is not necessary for water exchange in and out of the CSF, brain, or spinal cord In infants with HC, the increased half-time of D_2_O exchange was attributed to reduced ratio between ventricular surface area and volume Water exchange and CSF accumulation are separate phenomena
13	*Subjects:* (i) Severely impaired children with normal ventricular system (*n* = 4). (ii) Infants with HC (*n* = 3, two with non-communicating HC and one with communicating HC before CSF diversion). *Intervention:* D_2_O (0.4–2.4 mL/kg) injected to CV or CM or IV and analyzed using densitometry or mass spectrographic method from venous blood of SSS or antecubital vein, and CSF from CV, CM and lumbar SAS. *Outcome measures:* Estimation of ventricular exchange half-time of D_2_O, i.e., half-time of exchange from CSF to blood, and half-time of exchange from blood to CSF	Normal ventricular system: *In one child:* ventricular exchange half-time based on TBW (not blood samples) was ~5 min D_2_O given to ventricles is in equilibrium with total body water within about 3 h Hydrocephalus: In one infant with obstructed Sylvian aqueduct: 82% of ICV D_2_O absorbed to blood after 4 h (ventricular exchange half-time 100 min) Estimated ventricular exchange half-time after intravenous D_2_O based on TBW in HC cases: 80–180 min Appearance half-time after IV D_2_O (in two HC cases): 90–120 min D_2_O exchange half-life after intraventricular D_2_O in good agreement with D_2_O appearance half-time after intravenous D_2_O In one patient with non-communicating HC: D_2_O did not appear in lumbar CSF after it had appeared in blood, indicating that lumbar D_2_O was blood-borne	Speed of D_2_O exchange depends on ventricular size rather than on communicating CSF pathways. Obstruction of the Sylvian aqueduct does not prevent removal of D_2_O from the ventricular CSF, and the D_2_O exchange seems to proceed at the same rate whether administered intravenously or in the ventricle Exchange of D_2_O from the ventricular CSF to the blood stream is slower in the HC patients than the “normal” patients due to smaller exchange area-volume ratio in HC, i.e., ventricular exchange depends on “exchange area–volume ratio” which is low in HC CSF diversion had no effect on the amount or rate of D_2_O exchange from ventricles to blood, interpreted as CSF pressure in physiological ranges does not affect water exchange, which is rather controlled by the laws of diffusion
14	*Subjects:* Infant HC patients with removal of CP (*n* = 2). *Intervention:* D_2_O (dose: NR) injected IV and analyzed for D_2_O using densitometry or mass spectrographic method from CSF of CV. *Outcome measures:* Estimation of D_2_O exchange from blood to CSF before and after CP removal	No change in D_2_O exchange from blood to ventricular CSF after removal of CP CP removal did not affect D_2_O, ^24^Na, or ^131^I-albumin exchange	Concludes that CP has no major importance for CSF-blood water exchange, and that CSF accumulation should be considered separate from tracer ion exchange The author concludes that CSF should be considered as a mixture of many constituents that pass in and out of CSF spaces according to the physiological characteristics of each constituent
20	*Subjects:* Patients with psychiatric disease (depression) who were currently suffering (*n* = 11) or who had recovered (*n* = 10). *Intervention:* Tritiated water (T_2_O; 0.5 mCi) given IV and lumbar CSF analyzed for T_2_O using liquid scintillation counter. *Outcome measures:* Estimation of (i) Half-time where CSF concentration equals plasma concentration (“rate of entry from blood to CSF”), and (ii) transfer constant (min)	Differences in T_2_O entry half-time between individuals with current depression or who had recovered from depression Subjects with depressive disease: T_2_O entry half-time = 29.4 ± 6.0 min. T_2_O transfer constant = 0.021 ± 0.008 min Subjects recovered from depressive disease: T_2_O entry half time = 24.3 ± 4.7 min. T_2_O transfer constant = 0.025 ± 0.008 min	Entry of water from blood to CSF is altered in depression. Water exchange related to psychiatric disease
21	*Subjects:* Patients (aged 8–50 years) with no changes to the CSF spaces (*n* = NR). *Intervention:* T_2_O (30 µCi/kg), ^24^Na, ^36^Cl, ^42^K, ^32^P, ^82^Br, ^131^I-albumin administered IV and ventricular CSF and lumbar CSF analyzed for T_2_O using liquid scintillation counter (beta radiation, thin mica, and window Geiger–Mueller counter, measured for 8 h after injection). *Outcome measures:* Estimation of equilibrium between blood and ventricular CSF, and between blood and lumbar CSF (% of injected activity (normalized to the 15-min sample)), i.e., blood-to-CSF passage	Rapid passage of T_2_O from blood to ventricular CSF after IV injection, with equilibrium between blood and CSF after 1.5 h which lasts for the whole experiment (8 h) The other substances tested (^24^Na, ^36^Cl, ^42^K, ^32^P, ^82^Br, ^131^I-albumin) showed a completely different and slower pattern	The passage of T_2_O from blood to ventricular CSF shows a faster and different profile as compared with ions and other molecules Water passes from blood to ventricular CSF by a simple mechanism of diffusion Water enters the CSF freely, and equilibrium with blood is reached in a short time
28	*Subjects:* Healthy adult individuals (*n* = 12). *Intervention:* Ultra-long TE ASL MRI. *Outcome measures:* Assessment of water passage across blood vessels from blood to CP and SAS CSF; a time constant (*T*_blood → CSF_ (s)) describes the exchange of labeled water between the blood and CSF compartment	Mean transfer time from blood to CSF (*T*_blood → CSF_): CP: 60 s, SAS: 60 s, and WM: 80 s The CSF–ASL signal is fairly well distributed around the cortex, and not solely confined to the CP	Water exchange is widespread throughout the brain and not restricted to the CP Presence of blood–CSF water exchange sites around the cortex (SAS) due to AQP1 channels in pial vasculature?

AQP: aquaporin; CP: choroid plexus; CSF: cerebrospinal fluid; CM: cisterna magna; CV: cerebral ventricles; HC: hydrocephalus; ICV: intracerebroventricular; IV: intravenous; MRI: magnetic resonance imaging; NR: not reported; SAS: subarachnoid space; TBW: total body water.

### Direction and magnitude of CSF–blood water exchange

Bering first demonstrated that intravenous D_2_O in dogs resulted in rapid exchange with brain tissue (12–20 s) and CSF (3–8 min), with cisternal CSF equilibrating faster (~3 min) than ventricular CSF (~8 min).^12^ In monkeys, cisternal D_2_O likewise equilibrated rapidly with blood.^13^ Several studies across nonhuman species confirmed rapid water passage from ventricular CSF to blood,^17^ and from the subarachnoid space (SAS) to venous blood via pial arteries, with mean transit times of ~14 s (range 5–28 s).^18^

In humans (“healthy” individuals aged 6 months–87 years), Bering measured half-times for CSF compartments to reach blood D_2_O concentrations after intravenous administration, reporting: cisterna magna, 1.5–6 min, cerebral ventricles 2–37 min, and lumbar SAS 7–38 min.^12^ Migliore et al.^21^ later showed that T_2_O administered either intravenously or intraventricularly equilibrated between blood and CSF within ~1.5 h in individuals aged 8–50 years without CSF pathology.

Modern H_2_^17^O- and ASL–MRI studies corroborate this rapid bidirectional exchange. In rodents and humans, blood-to-CSF delivery at the choroid plexus and cortical SAS occurs with characteristic time constants of ~1 min.^26,28^ Using ultra-long echo time ASL–MRI, Petitclerc et al.^28^ quantified blood-to-CSF transfer times of ~60 s for both choroid plexus and SAS, and ~80 s in white matter. Notably, CSF–ASL signal was broadly distributed around the cortex, indicating that water exchange is not confined to the choroid plexus.

Collectively, these findings show that water exchange between blood and CSF is rapid, continuous, and bidirectional, rather than a unidirectional process of CSF “production” and “absorption.”

The reported (semi-)quantitative estimates suggest that the total volume of water exchanged between blood and CSF is large relative to the CSF pool (Supplementary Table 3 and Table 3). Based on isotopic and MRI-derived half-times, we estimate the effective flux to about 0.5–2.0 mL/min, which would be sufficient to renew the entire CSF water content (~150 mL) several times per day.

### Anatomical distribution of exchange along the craniospinal axis

Evidence for blood–CSF water exchange is found at multiple interfaces extending from the cerebral ventricles to the lumbar SAS. At the choroid plexus, ASL and H_2_^17^O MRI in rodents and humans demonstrate rapid blood-to-CSF water transfer, closely linked to perfusion.^26,28^ Across ventricular ependyma and periventricular capillaries, classical isotope studies indicate diffusion-dominated exchange with permeability coefficients in the range of 10^−4^–10^−3^ cm/s.^12,16^ At pial and cortical vessels, water moves from CSF to venous blood within seconds, independently of ion transport,^18^ indicating a highly permeable interface. Evidence of spinal water exchange comes from early D_2_O studies in which tracer appeared in lumbar CSF even when cranial CSF pathways were obstructed,^12,13^ demonstrating that exchange also occurs locally along the spinal SAS.

Integrating data across compartments suggests that total CSF–blood water exchange is distributed approximately as follows: ~25% via the choroid plexus, ~20% via the ventricular wall, ~25% via cortical and pial surfaces, and ~30% via the spinal SAS (Table 3). Exchange is faster in cisternal and spinal compartments than within the ventricles, reflecting greater surface area and vascular density. These findings suggest that blood–CSF water turnover is a system-wide process rather than a localized secretion, and that the craniospinal compartment operates as a continuous diffusion interface between vascular and CSF water pools.

**Table 3.** Estimated water exchange between blood and CSF across craniospinal sites.

Anatomical site	Representative references	Typical half-time (*t*½)	Approximate permeability/flow	Estimated contribution to total exchange	Dominant direction of flux	Mechanistic features
CP	26–28	~1 min (blood → CSF turnover time)	0.5–1 mL/min (human-equivalent)	~25%	Blood → CSF	AQP1-rich epithelium; perfusion-dependent; modulated by anesthetics
Ventricular ependyma/periventricular capillaries	12–14, 19	5–30 min	Permeability 10^−4^–10^−3^ cm/s	~20%	Bidirectional (CSF ↔ blood)	Diffusion-dominated; AQP4-mediated; pressure-sensitive
Cisternal and cortical subarachnoid space (pia/surface vessels)	17, 18, 21	Seconds–few minutes	Very high; effective half-time ≈ 10 s	~25%	CSF → blood (rapid clearance)	Pial microvasculature transports water but not ions
Spinal subarachnoid space	12, 13	3–7 min	Not quantified; similar to cisternal	~30%	Bidirectional	Local capillary exchange independent of cranial communication
Whole craniospinal system (aggregate)	Combined isotopic + MRI data	–	0.5–2 mL/min total	100%	Bidirectional; region-specific	Diffusive exchange across multiple vascular surfaces; renews total CSF water several times per day

CP: choroid plexus.

Tracer studies also indicate that the choroid plexus removal does not substantially alter the appearance of D_2_O, ^24^Na, or ^131^I-albumin in CSF after intravenous administration,^12,14^ further supporting a distributed exchange system.

### Driving forces governing water exchange

Experimental manipulation of pressure, osmolarity, and hormonal state suggests that the direction of blood–CSF water flux is dynamic and locally regulated. Early isotope studies identified diffusion as the principal mechanism of water transfer across blood–CSF interfaces,^12,15^ and Migliore et al.^21^ likewise attributed blood-to-CSF water movement in humans to simple diffusion.

Hydrostatic forces also contribute: elevations in intraventricular pressure increased T_2_O efflux from ventricles to blood,^15^ whereas increased choroid plexus perfusion or arterial pressure enhanced blood-to-CSF inflow.^26^ Osmotic gradients can reverse the direction of movement, as demonstrated by the introduction of hyperosmolar mannitol into CSF, which shifted net flux toward blood.^16^ Hormonal modulation was evident when vasopressin increased ependymal permeability and accelerated CSF clearance.^19^

Evidence was given that permeability is adjustable: deletion or pharmacologic modulation of AQP4 altered the rate of water exchange without changing its direction, indicating that aquaporins regulate conductivity rather than net flux polarity.^22–24^

Table 4 summarizes studies demonstrating that water exchange across blood–CSF interfaces occur largely independently of ionic or macromolecular transport. Bering observed distinct exchange patterns for D_2_O, ^24^Na, ^42^K, and ^131^I-albumin in dogs^14^: water moved rapidly, whereas ions and proteins crossed more slowly. Similar findings were reported in monkeys: after cisternal injection, T_2_O equilibrated with blood within ~10 min, ^22^Na required ~45 min, and ^131^I-albumin moved even more slowly, likely influenced by sagittal sinus absorption.^17^ In goats, ventricular permeability coefficients differed markedly (T_2_O: 1.7 ± 0.5 cm^2^/min; ^24^Na: 0.18 ± 0.01 cm^2^/min; ^42^K: 0.80 ± 0.13 cm^2^/min for).^15^ In dogs and cats, T_2_O rapidly appeared in venous blood after subarachnoid or ventricular administration, while ^22^Na and ^3^H-inulin exhibited markedly slower movement.^16,18^ In humans, Migliore et al.^21^ found that T_2_O exchanged rapidly, whereas other tracers (^24^Na, ^36^Cl, ^42^K, ^32^P, ^82^Br, ^131^I-albumin) showed delayed and heterogeneous kinetics.

**Table 4.** Studies comparing water and ionic tracers.

Reference	Design	Findings	Interpretation
*Classical tracer studies directly comparing H_2_O and ionic tracers: water equilibrates far faster than ions, indicating independent transport*			
12, 14	Intravenous and intraventricular D_2_O compared with ^24^Na, ^42^K, and ^131^I-albumin in dogs and humans	(i) D_2_O equilibrated between blood and CSF within minutes, whereas ions required tens of minutes to hours. (ii) Removal of the choroid plexus did not alter D_2_O exchange but did affect solute kinetics	Water crosses the blood–CSF interface freely and rapidly, by diffusion independent of ion transport These papers are historically the most explicit demonstration that water movement is uncoupled from ionic flux
17	Monkeys received cisternal injections of T_2_O, ^22^Na, and ^131^I-albumin; radioactivity tracked in venous and arterial blood	(i) T_2_O appeared in blood within 5–10 min. (ii) ^22^Na equilibrated only after ≈45 min; ^131^I-albumin much later	Strong evidence that CSF-to-blood water flux through vascular walls is independent of Na^+^ or protein transport
18	Dogs with a compartmentalized subarachnoid space received T_2_O and ^22^Na injections; venous blood sampled before and after pial vessel removal	(i) T_2_O appeared in venous blood within seconds (mean 14 s). (ii) No detectable ^22^Na appeared in blood during the same interval	Pial vessels transport water but not Na^+^ from CSF to blood—perhaps the clearest experimental proof of solute-independent water flux across the blood–CSF barrier
15	Ventriculo-cisternal perfusion in goats with T_2_O, ^24^Na, ^42^K	Permeability coefficients: (i) T_2_O ≈ 1.7 cm^3^/min. (ii) ^24^Na ≈ 0.18 cm^3^/min. (iii) ^42^K ≈ 0.80 cm^3^/min	Diffusion dominates water loss from ventricular CSF, and H_2_O crosses far more readily than ions, again supporting independent transport
21	Compared intravenous T_2_O with ^24^Na, ^36^Cl, ^42^K, ^32^P, ^82^Br, ^131^I-albumin in humans	(i) T_2_O equilibrated between blood and CSF within ~1.5 h. (ii) All other tracers were markedly slower or incomplete	Water diffuses freely between blood and CSF, whereas ionic and protein tracers do not—direct human evidence for independent solvent exchange
*Ependymal and parenchymal absorption experiments: ependyma freely permeable to water, not to solutes*			
16	Cats infused with T_2_O and ^3^H-inulin into ventricles or cortical subarachnoid space	T_2_O equilibrated rapidly across ependyma and pia; ^3^H-inulin moved slowly and bidirectionally	Ependyma provides no barrier to water, whereas solute exchange is limited—direct confirmation of decoupled solvent and solute permeability
*Modern experimental imaging evidence confirming rapid, solute-independent water flux: AQP4 enables selective water flux without ions*			
22, 24	JJ-vicinal coupling ^17^O-MRI with H_2_^17^O in wild-type vs AQP-knockout mice	(i) AQP4 deletion markedly reduced H_2_^17^O influx into CSF; AQP1 deletion did not. (ii) No parallel solute movement measured	AQP4-dependent transcellular water diffusion operates independently of ionic transport or osmotic bulk flow
23	JJ vicinal coupling proton exchange MRI and IV H_2_^17^O	Decreased H_2_^17^O uptake and retention in AQP4-KO mice despite intact solute barriers	Confirms water-specific transport via AQP4
*Modern human imaging evidence confirming rapid, solute-independent water flux, consistent with diffusion/AQP transport*			
28	Ultra-long-TE ASL–MRI in humans	Blood-to-CSF water transfer constant ≈60 s at both choroid plexus and subarachnoid space; signal distributed cortex-wide, not limited to CP	Rapid brain-wide water exchange consistent with AQP-mediated diffusion, not with bulk solute-driven filtration

ASL: arterial spin labeling.

Together, the reported results indicate that hydrostatic, osmotic, and molecular gradients jointly determine the local balance between inflow and outflow, making net water transport highly context dependent. The pronounced dissociation between water and solute flux may be most parsimoniously explained by AQP-mediated diffusion through selective, high-permeability membranes, allowing water to cross rapidly while restricting ionic and macromolecular passage.

### Molecular and cellular mechanisms of water transfer

Aquaporin water channels provide the molecular basis for rapid water exchange between blood and CSF. AQP1, expressed on choroid plexus epithelial cells, facilitates trans-epithelial water movement from blood to ventricular CSF. AQP4, densely localized to astrocytic endfeet at perivascular, ependymal, and pial surfaces,^30^ governs permeability on the brain side of the vascular wall. Knockout or mis-localization of AQP4 reduces water exchange by ~20%–40% in optical and MRI studies,^24^ confirming its rate-limiting role.

Three pharmacologic modulators demonstrated effects on water transport:

i. *Arginine vasopressin (AVP)* enhances CSF-to-blood water efflux. In cats, AVP increased the T_2_O distribution volume, raised ependymal permeability, and shortened capillary transfer half-time during ventriculo-cisternal infusion experiments.^19^ The net effect was accelerated removal of water from ventricular CSF into brain tissue and blood (enhanced CSF-to-blood absorption),^19^ consistent with increased transcellular permeability at the CSF–brain interface.
ii. *Aquaporin-4 (AQP4) modulation* alters parenchymal water flux and affects water transport across the BBB and BCSFB. In AQP4-knockout mice, H_2_^17^O penetration into third ventricle CSF after intravenous administration was significantly reduced, whereas AQP1-knockout mice showed no difference, implicating AQP4 in blood-to-CSF water entry.^24^ Intravenous H_2_^17^O combined with ^17^O-MRI showed reduced brain water signal in AQP4-knockout mice, reflecting impaired BBB water transport.^23^ Conversely, the AQP4 facilitator TGN-073 enhanced cortical water flux and increased interstitial fluid (ISF) turnover, interpreted as faster ISF-to-perivascular/blood exchange.^25^ Notably, CSF water transport itself was not measurably increased, suggesting a predominantly parenchymal/perivascular mode of action rather than enhanced blood → CSF entry.
iii. *Anesthetic state* modulates blood-to-CSF delivery at the choroid plexus. In rats, continuous ASL–MRI demonstrated that BCSFB-mediated water delivery from arterial blood to ventricular CSF was higher under isoflurane than dexmedetomidine anesthesia, with corresponding differences in arterial transit times.^26^ Although not a therapeutic modulation, this provides a clear proof-of-principle that pharmacology can tune blood-to-CSF water transport at the choroid plexus.

Together, these findings indicate that water movement across blood-to-CSF interfaces is a dynamic and regulatable process, not a fixed passive property. Because water crossing the capillary wall must pass through sequential barriers, the endothelium and the AQP4-rich astrocytic endfeet, changes in AQP4 expression or polarization alter the overall hydraulic conductivity between blood and CSF, even though AQP4 is not located within the endothelial membrane itself.

### Impact of disease

Several studies identified alterations in blood–CSF water exchange associated with neurological and psychiatric conditions.

In APP–PS1 transgenic mice (a model of Alzheimer’s disease), intravenous H_2_^17^O combined with JJ vicinal coupling proton exchange MRI (JJVCPE–MRI) demonstrated reduced water flux into third-ventricle CSF, suggesting impaired blood-to-CSF water entry early in the disease course.^22^ In spontaneously hypertensive rats, ASL–MRI revealed a 35.8% reduction in BCSFB-mediated ventricular water delivery compared with normotensive controls (14.4 ± 1.92 vs 9.22 ± 1.20 µL/min).^27^ Conversely, in another Alzheimer’s mouse model (sXTg), the same group reported increased choroid plexus-mediated ventricular water delivery,^29^ highlighting model-specific differences in CP perfusion and water flux.

Early D_2_O studies in hydrocephalus also demonstrated altered exchange. Bering reported markedly prolonged half-times for D_2_O entry into the ventricles (60–245 min) and lumbar SAS (9–36 min) in hydrocephalic infants compared with non-hydrocephalic children and adults (ventricles, 2–37 min; lumbar SAS, 7–38 min).^12^ He attributed the slowed exchange to a reduced ventricular surface-area-to-volume ratio. Importantly, ventricular wall exchange persisted despite Sylvian aqueduct obstruction and occurred at similar rates regardless of whether D_2_O was administered intravenously or intraventricularly.^13^ In one case of non-communicating hydrocephalus, D_2_O appeared in lumbar CSF only after its appearance in blood, indicating a blood-borne route.^13^ Diverting CSF did not change ventricular-to-blood D_2_O exchange rates, leading Bering to conclude that physiological CSF pressure has little influence on water exchange, which is primarily diffusion-driven.^13^

Altered water exchange has also been observed in psychiatric disease. Coppen et al.^20^ administered T_2_O intravenously and intrathecally in patients with major depression. Individuals with current depression exhibited slower blood-to-CSF transfer (half-time 29.4 ± 6.0 min; transfer constant 0.021 ± 0.008 min) than recovered patients (24.3 ± 4.7 min; 0.025 ± 0.008 min), suggesting that impaired CSF–blood water exchange may accompany mood disorders.

## Discussion

**Figure 2.**
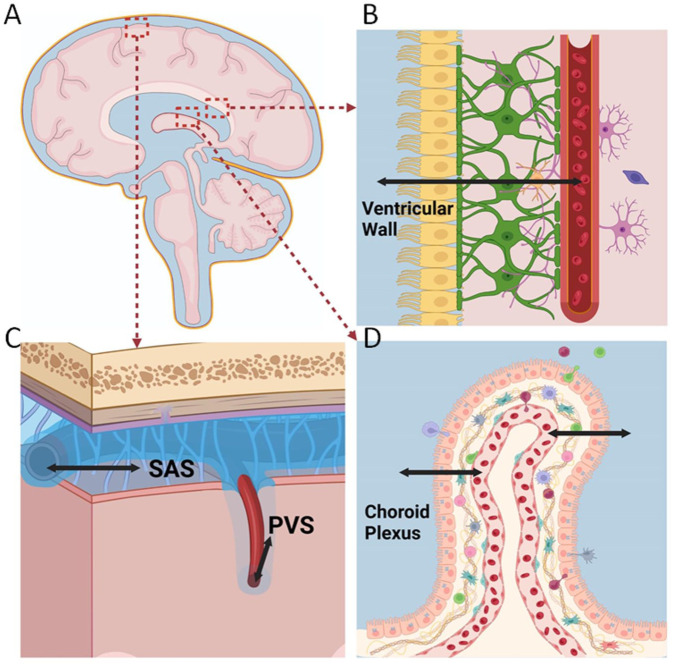
Locations for water passage between CSF and blood at blood–CSF-barriers. (a) The blood–CSF-barriers examined in the included reviews include, (b) ventricular wall, that is, lined by the ependymal cells, (c) the pial arteries in SAS and the brain parenchyma (that corresponds to blood vessels within the parenchymal perivascular spaces), and (d) the choroid plexus. Illustration: Dr. Cesar Luis Vera Quesada, Oslo University Hospital, University of Oslo. SAS: subarachnoid space.

This systematic review identified a small but informative body of literature on blood–CSF water exchange spanning more than 70 years and comprising 18 studies—15 in animals, six in humans, and three that included both. Collectively, these studies show that water moves rapidly and bidirectionally across the blood–CSF barriers, including the vasculature of the choroid plexus, ventricular walls, subarachnoid space, and perivascular compartments within the parenchyma (Figure 2). Exchange occurs predominantly by diffusion and is modulated by hydrostatic gradients and surface area. Contrary to the traditional view,^7^ the choroid plexus appears to play a more limited role in water entry into the CSF than previously assumed. Importantly, water transfer can occur independently of solute movement, including ions (Na^+^, K^+^) and larger molecules such as inulin and albumin. Evidence for molecular regulation is more limited but highlights the roles for AQP4 and arginine vasopressin in modulating permeability.

As summarized in Tables 1 and 2, the methods for assessing blood–CSF water exchange have evolved considerably over time. Early invasive techniques using D_2_O and T_2_O provided quantitative estimates of exchange but offered limited spatial and temporal resolution. More recent non-invasive approaches—including ASL–MRI and ^17^O-sensitive MRI—allow anatomical mapping and mechanistic assessment of blood–CSF water transfer, although they currently lack the direct quantitative precision of classical tracer studies.

### Reinterpreting “production” and “absorption” as bidirectional equilibrium

Across animal and human studies using D_2_O, T_2_O, and H_2_^17^O tracers, water was found to move rapidly and bidirectionally between blood and CSF. Importantly, this reciprocal exchange persisted even after choroid plexus isolation, directly challenging the classical view that CSF is formed and absorbed at discrete, anatomically fixed sites. Instead, the apparent “production” or “absorption” observed under physiological or pathological conditions reflects transient dominance of inflow or outflow driven by local hydrostatic, osmotic, or permeability gradients. CSF homeostasis is therefore best conceptualized as a steady-state equilibrium maintained by distributed, bidirectional water flux.

The traditional “third circulation” model, in which CSF is produced primarily by the choroid plexus and absorbed into the dural sinus veins to establish a unidirectional bulk flow,^2^ is inconsistent with evidence showing water entry and exit at multiple CNS interfaces. While some authors have argued that widespread vascular exchange precludes the existence of net CSF flow, claiming, for example, that no net movement occurs through the Sylvian aqueduct,^4^ this position contradicts phase-contrast MRI studies demonstrating net CSF (water) flow through both the aqueduct and the cranio-cervical junction.^31,32^ Thus, even though water may enter the CSF from the entire CNS vasculature, local pressure gradients can still generate regions of net directional flow within the ventricular and subarachnoid spaces.

The long-standing perspective that CSF is a single, uniform entity produced almost exclusively by the choroid plexus can be partly traced to methodological conventions. A key example is the ventriculo-cisternal perfusion technique,^15^ which estimates “CSF production” by measuring the clearance of inulin, a tracer molecule of 3–5 kDa (with a broader range of 1–10 kDa). Because this method quantifies the removal of a solute, it implicitly treats CSF as a homogeneous fluid and overlooks the fact that its major constituent—water—has distinct exchange pathways and kinetics. As evidenced by the studies in this review, CSF water derives from multiple sources throughout the CNS, not solely the choroid plexus, and diseases can selectively alter these local water-exchange mechanisms.

Thus, referring to “CSF production” without acknowledging the diverse origins and behaviors of its solvent and solute components oversimplifies the physiology. A more nuanced framework that recognizes the heterogeneous composition of CSF, its multiple entry routes, and the regulatable nature of water exchange provides a more accurate foundation for understanding both normal CSF dynamics and their alterations in disease.

### Anatomical distribution of exchange along the craniospinal axis

Water exchange occurs throughout the craniospinal system, involving the choroid plexus,^26,28,29^ ventricular ependyma,^14–16^ cortical pial vessels,^17,18^ perivascular and parenchymal interfaces,^22–24^ and the spinal subarachnoid space.^12,13^ The consistency of tracer kinetics and MRI findings across these interfaces indicates that the CSF system functions as a distributed exchange network rather than a linear production-absorption circuit. Both spinal and cortical vessels contribute substantially to total water flux, suggesting that the entire perivascular continuum participates in maintaining CSF–blood water equilibrium.

Several findings highlight the importance of the spinal compartment. Intravenous D_2_O appeared in the lumbar SAS at similar rates in individuals with intact cranial communication and in those with isolated lumbar CSF spaces,^12^ implying that water entry into lumbar CSF occurs locally and independently of cranial pathways. Consistent with this interpretation, modern MRI studies have repeatedly demonstrated net upward CSF water flow at the cranio-cervical junction in supine individuals.^31,32^ Moreover, the average time for a lumbar administered CSF tracer to reach the cisterna magna was 20 ± 23 min,^33^ indicating rapid CSF flow in the cranial direction. Given the increased hydrostatic pressure gradients in the upright posture, spinal contributions to CSF water entry are expected to be even more prominent when standing.

Across these regions, the apparent direction of CSF–blood water movement is not fixed but reflects the balance of local hydrostatic, osmotic, and permeability conditions. Elevated arterial or choroid plexus perfusion pressure enhances blood-to-CSF inflow, whereas increased ventricular pressure, CSF hyperosmolarity, or vasopressin stimulation favors CSF-to-blood efflux. Because these gradients vary spatially and dynamically, the craniospinal system operates as a bidirectional equilibrium, in which traditional notions of “production” and “absorption” reflect transient, region-specific biases rather than anatomically segregated processes.

### Mechanistic basis of water exchange

Quantitative data from early tracer work and modern MRI methods converge on the conclusion that water exchange between blood and CSF is rapid, continuous, and large relative to total CSF volume, occurring on the order of seconds to minutes rather than hours. Bering’s early D_2_O studies showed that CSF tracer disappearance closely paralleled declines in blood concentrations, consistent with ongoing equilibration rather than unidirectional “production” or “absorption.”

Diffusion appears to be the principal mechanism driving this exchange. Partial-pressure and concentration gradients of H_2_O across vascular, ependymal, and pial interfaces provide sufficient chemical potential to support substantial flux, even in the absence of solute movement. Small hydrostatic differences further augment transcapillary flow due to the finite hydraulic conductivity of brain microvessels. For example, experimentally increasing intraventricular pressure enhanced T_2_O efflux,^15^ demonstrating a pressure-dependent component superimposed on diffusion. Thus, what is traditionally interpreted as “production” or “absorption” likely reflects regional differences in diffusion rates, surface area, and local driving forces rather than directional flow in a single circuit.

At any given site, the net direction of water movement is set by the interplay of hydrostatic, osmotic, and permeability gradients. Elevated ventricular pressure favors CSF-to-blood movement, whereas increased arterial or choroidal perfusion promotes blood-to-CSF entry. Hyperosmolar CSF enhances absorption; hypo-osmolar plasma enhances entry. Vasopressin and AQP4 regulation alter effective permeability and thus shift the balance accordingly. These regionally variable and dynamic gradients explain how the system maintains stable CSF volume despite fluctuating pressures and osmolarities.

The short half-times for water equilibration (seconds to minutes) contrast sharply with those for ions (Na^+^, K^+^) or macromolecules (inulin, albumin), which require tens of minutes to hours (Table 4). This dissociation demonstrates that water transport is not obligatorily coupled to solute flux. CSF and interstitial water are in near-continuous exchange with capillary and perivascular water, forming a dynamic fluid continuum rather than a slowly circulating, isolated compartment. For instance, rapid water transfer at pial arteries occurred without accompanying Na^+^ exchange.^18^ This implies that CSF composition is not uniform and likely varies according to different sites of exchange—a view supported by documented differences between lumbar and ventricular CSF.^34^

The classical Starling principle that couples solvent and solute transport,^9^ does not fully apply to brain microvessels. Instead, selective hydraulic permeability and aquaporin-mediated water diffusion allow rapid water passage across endothelial, ependymal, and glial barriers even when ion gradients are minimal. AQP enables single-file water movement along osmotic or pseudo-osmotic gradients without solute transfer, resolving the apparent paradox posed of solvent-solute uncoupling. Consistently, AQP4-knockout animals show reduced H_2_^17^O penetration into CSF and brain interstitial tissue, confirming its key role (see Tables 1 and 2^22–24^). AQP1 in the choroid plexus facilitates trans-epithelial water flow but appears less essential for whole-system CSF–blood exchange. The intracellular and extracellular water phases of the CNS form a nearly continuous medium, allowing diffusive exchange across ependyma, pia, and perivascular spaces even when solute diffusion is restricted.

Hormonal and physiological state further modulate exchange. Vasopressin increased ependymal permeability and enlarged the T_2_O distribution space in cats,^19^ likely via AVP-AQP4 signaling, while anesthetic state (isoflurane vs dexmedetomidine) altered choroid plexus blood-to-CSF transfer on ASL–MRI. These observations reinforce that water flux across the BCSFB is a regulated physiological process, not a fixed passive property.

### Molecular and cellular mechanisms of water transfer

The reviewed studies provide consistent evidence that AQP4 is the principal molecular regulators of water exchange between blood, interstitial fluid, and CSF. AQP4 is densely expressed in astrocytic endfeet along perivascular, subpial, and ependymal surfaces, that is, the membranes that interface with CSF and the microvasculature.^30^ In AQP4-knockout mice, H_2_^17^O influx into ventricular CSF was significantly reduced, whereas AQP1 deletion in choroid plexus had no measurable effect,^24^ indicating that AQP4, not AQP1, mediates the dominant water pathway across the CNS fluid barriers.

Structurally, water transfer from blood to the interstitial space of the brain parenchyma requires passage through three layers: (i) The endothelial membrane. (ii) Basement membrane. (iii) AQP4-rich astrocytic endfeet. These layers act as serial hydraulic resistors that together determine the effective permeability of the brain-CSF surface:

$$\begin{array}{l} Total\;hydraulic\;resistance\;=\;R_{1}\;(endothelium)\;+\;\\ \;\;\;\;\;\;\;\;\;\;\;\;\;\;\;\;\;\;\;\;\;\;\;\;\;\;\;\;\;\;\;\;\;\;\;\;\;\;\;\;\;\;\;\;\;\;\;\;\;\;\;\;\;\;\;\;\;\;\;R_{2}\;(basal\;lamina)\;+\;\\ \;\;\;\;\;\;\;\;\;\;\;\;\;\;\;\;\;\;\;\;\;\;\;\;\;\;\;\;\;\;\;\;\;\;\;\;\;\;\;\;\;\;\;\;\;\;\;\;\;\;\;\;\;\;\;\;\;\;\;R_{3}\;(AQP4\;endfoot) \end{array}$$

Ultrastructural studies^35,36^ show AQP4 clustered on both the perivascular and neuropil-facing surfaces of astrocytic endfeet, though less polarized to the perivascular membrane in humans than mice.^36^ In vivo imaging and genetic models^37,38^ demonstrate that deletion or loss of AQP4 polarization markedly reduces fluid and solute flux between perivascular and interstitial compartments. These data support a transcellular “hydraulic bridge” model, in which water entering the perivascular basal lamina crosses the endfoot cytoplasm via paired AQP4 domains, allowing rapid exchange while maintaining ionic segregation. This explains how water—but not ions or macromolecules—can move freely between blood and CSF.

Regulatory mechanisms further tune this equilibrium. Vasopressin increases ependymal permeability and accelerates CSF clearance, likely via AQP4-dependent pathways, while anesthetic agents modulate blood-to-CSF inflow at the choroid plexus in ASL–MRI studies. Together, these findings show that the molecular machinery governing water transfer is not static but dynamically regulated by physiological and pharmacological states.

### Glymphatic transport versus blood–CSF water exchange

How do the present observations relate to the glymphatic framework? At first glance, the glymphatic model, characterized by net CSF influx along periarterial spaces and efflux along perivenous pathways,^38^ appears inconsistent with the bidirectional transvascular water exchange demonstrated in the reviewed literature. However, these processes operate at different spatial and temporal scales and are best understood as complementary rather than contradictory.

Glymphatic flow reflects bulk convective transport of fluid and solutes within the perivascular continuum.^38^ In contrast, the tracer and MRI data reviewed here quantify microscopic, bidirectional exchange of water molecules across endothelial and astrocytic membranes. Continuous molecular-level exchange does not preclude a net convective bias; rather, both processes can coexist. In this framework, AQP4 provides the low-resistance transcellular pathway that links microvascular water permeability to macroscopic glymphatic transport.

Converging evidence supports this integrated model: AQP4 deletion slows glymphatic tracer clearance,^38,39^ and H_2_^17^O studies show that AQP4 also mediates rapid bidirectional water exchange across the neurovascular unit.^23,25^ Human evidence includes patients with the dementia subtype idiopathic normal pressure hydrocephalus (iNPH), who present with loss of perivascular AQP4^40,41^ and impaired glymphatic function shown by intrathecal contrast-enhanced MRI.^42,43^

Thus, the same perivascular channel underlies both microscopic exchange and macroscale convective flow, with the prevailing driving forces (pressure, osmolarity, or permeability) determining whether net movement occurs. Glymphatic flow describes the direction of bulk movement, whereas CSF–blood exchange explains how water is continuously renewed and redistributed.

Together, these findings suggest that the CSF–blood exchange network provides the hydraulic substrate on which the glymphatic circulation operates. Factors that alter local pressure gradients, osmolarity, endothelial permeability, or AQP4 function are therefore expected to modulate both microscale water exchange and macroscale solute clearance in the brain.

### Blood–CSF water exchange in disease

Pathological conditions modify the regulation and efficiency of blood–CSF water exchange. Experimental studies show reduced water flux in models of hypertension^27^ and Alzheimer’s disease,^29^ whereas enhanced exchange has been observed in certain early Alzheimer’s stages,^29^ possibly reflecting compensatory choroid plexus hyperperfusion. In humans, altered T_2_O entry into CSF in patients with depression suggests that psychiatric disease may also involve subtle disturbances in water homeostasis.

The available literature on disease-related changes remains limited but informative. Bering’s investigations of infantile hydrocephalus demonstrated markedly slowed blood-to-CSF water exchange, implicating the surface-area-to-volume ratio of the ventricles as a key determinant of water movement.^13^ Notably, water absorption across the ventricular wall persisted even when the Sylvian aqueduct was occluded, indicating that exchange can occur independently of intact CSF pathways. Furthermore, choroid plexus removal did not alter water transfer between blood and CSF,^14^ reinforcing the concept of a distributed exchange system.

Other pathological models provide converging evidence. Experimental hypertension reduces blood-to-CSF delivery at the choroid plexus,^27^ and both preclinical and transgenic Alzheimer’s models show altered BCSFB water transport.^22,29^ These disturbances may reflect impaired perfusion, vascular stiffening, or inflammatory changes affecting barrier permeability.

Loss of AQP4 polarization or genetic deletion further decreases water turnover, contributing to impaired interstitial and CSF clearance. In hydrocephalus, reduced ependymal permeability slows CSF-to-blood efflux, promoting ventricular expansion. Although initial dilatation temporarily increases the surface area available for exchange, progressive enlargement ultimately reduces the surface-area-to-volume ratio, limiting compensatory capacity. Hypertension and neurodegeneration disease may similarly alter local pressure, osmotic gradients, or membrane permeability, shifting the equilibrium toward net retention or depletion of CSF water.

### Limitations

This review is subject to several notable limitations. First, the literature on blood–CSF water exchange is sparse and relies heavily on older studies that used invasive tracer methods with limited spatial and temporal resolution. Although foundational, these techniques may not adequately capture the complexity of rapid, regionally variable water exchange that modern imaging approaches can reveal.

Second, the majority of available data derive from animal models, with relatively few studies in humans. This limits the direct translational relevance of findings and underscores the need for more human-focused research.

Third, significant gaps remain in our understanding about the molecular and pharmacological mechanisms that regulate blood–CSF water flux, particularly in neurological disease. Continued development of advanced, non-invasive imaging and molecular approaches will be essential to clarify these mechanisms and to characterize how they are altered in pathology.

## Conclusions

Together, the findings of this systematic review indicate that CSF water exchange is a distributed, diffusion-dominated process occurring across multiple interfaces rather than a unidirectional secretory–absorptive system centered on the choroid plexus. Water exchanges continuously between blood, interstitial fluid, and CSF along the entire craniospinal axis, driven by local gradients in hydrostatic pressure, surface area, permeability, and osmolality. This exchange can occur independently of solute transport, underscoring the need to distinguish CSF water dynamics from solute circulation. Recognizing CSF as a heterogeneous fluid, whose water and solute components follow distinct anatomical and molecular pathways, fundamentally reframes classical notions of “production” and “absorption” as manifestations of transient, region-specific biases in a broader bidirectional equilibrium. AQP4 provides the principal low-resistance pathway linking vascular, glial, and ependymal interfaces, while disease states such as hydrocephalus, hypertension, and neurodegeneration likely disrupt this finely regulated hydraulic network. Despite these insights, contemporary evidence remains limited, and the molecular and pharmacological determinants of blood–CSF water exchange are poorly understood. Future work should integrate quantitative radiotracer imaging, high-resolution multimodal MRI, and molecular perturbations to map regional water fluxes, clarify spinal contributions, and determine how these processes are altered in neurological disease-ultimately informing therapeutic strategies that target aquaporin function or local fluid gradients to restore CSF homeostasis.

## Supplemental Material

sj-pdf-1-jcb-10.1177_0271678X251413926 – Supplemental material for Water exchange across the blood–CSF barrier: A systematic review Supplemental material, sj-pdf-1-jcb-10.1177_0271678X251413926 for Water exchange across the blood–CSF barrier: A systematic review by Trine Hjørnevik and Per Kristian Eide in Journal of Cerebral Blood Flow & Metabolism
