# Supplementary material for: Water exchange across the blood–CSF barrier: A systematic review
Source: J Cereb Blood Flow Metab. 2026 Feb 7:0271678X251413926. Online ahead of print. doi: 10.1177/0271678X251413926 (PMC12882841; doi:10.1177/0271678X251413926)
Supplement: sj-pdf-1-jcb-10.1177_0271678X251413926 – Supplemental material for Water exchange across the blood–CSF barrier: A systematic review [file sj-pdf-1-jcb-10.1177_0271678X251413926.pdf]

Supplementary Material

Water Exchange Across the Blood-CSF Barrier: A Systematic Review

Trine Hjørnevik, PhD[1,2,3], Per Kristian Eide, MD, PhD[2,3,4*],

[1]*Department of Physics and Computational Radiology, Oslo University Hospital, Oslo, Norway.*
[2]*Institute of Clinical Medicine, Faculty of Medicine, University of Oslo, Oslo, Norway.*
[3]*KG Jebsen Centre for Brain Fluid Research, University of Oslo, Oslo, Norway*
[4]*Department of Neurosurgery, Oslo University Hospital – Rikshospitalet, Oslo, Norway.*

***Corresponding author:**
Professor Per Kristian Eide, MD PhD
Department of Neurosurgery
Oslo University Hospital - Rikshospitalet
Pb 4950 Nydalen,
Phone: +47 91649419
Fax: +47-23074310
N-0424 Oslo, Norway
p.k.eide@medisin.uio.no

Content

Supplementary Table 1. Systematic Review Search Protocol

Supplementary Table 2. References

Supplementary Table 3. Magnitude estimates

Supplementary Table 1. Systematic Review Search Protocol

Sources used for the search:

**Database**	**April 23rd 2025**
MEDLINE (Ovid)	122
Embase (Ovid)	216
Scopus (Elsevier)	229
Number of records before deduplication:	567
Number of duplicates removed	256
Number of records after deduplication:	311

All searches were performed on 23 April 2025 by Academic Librarian Hilde Strømme, University of Oslo Library of Medicine and Science.

MEDLINE

Ovid MEDLINE(R) ALL <1946 to April 21, 2025>

1	Cerebrospinal Fluid/ or (neurofluid* or csf or ((cerebrospinal* or brain or cranial or spinal) adj3 (fluid* or liquid* or liquor))).tw,kw,kf.	196185
2	(water adj3 (exchange or transport* or flow or produ* or dynamics or kinetics)).tw,kw,kf.	48937
3	exp Blood/ or exp Blood Vessels/ or Endothelium, Vascular/ or Capillary Permeability/ or exp Cerebrovascular Circulation/ or (blood or vascula* or vein* or venous or arter* or capillar*).tw,kw,kf.	5082402
4	1 and 2 and 3	122

Embase

Embase Classic+Embase <1947 to 2025 April 21>

1	Cerebrospinal Fluid/ or (neurofluid* or csf or ((cerebrospinal* or brain or cranial or spinal) adj3 (fluid* or liquid* or liquor))).tw,kw,kf.	333662
2	(water adj3 (exchange or transport* or flow or produ* or dynamics or kinetics)).tw,kw,kf.	54706
3	exp blood/ or exp blood vessel/ or vein endothelium/ or exp blood vessel permeability/ or exp brain circulation/ or (blood or vascula* or vein* or venous or arter* or capillar*).tw,kw,kf.	8327034
4	1 and 2 and 3	216

Scopus

TITLE-ABS-KEY ((neurofluid* OR csf OR (
(cerebrospinal* OR brain OR cranial OR spinal) W/2 (fluid* OR liquid* OR liquor))) AND
(water W/2 (exchange OR transport* OR flow OR produ* OR dynamics OR kinetics)) AND
(blood OR vascula* OR vein* OR venous OR arter* OR capillar*))

229 hits

Supplementary Table 2. References

(1-18)

1. Perera C, Cruz R, Shemesh N, Carvalho T, Thomas DL, Wells J, et al. Non-invasive MRI of blood-cerebrospinal fluid-barrier function in a mouse model of Alzheimer's disease: a potential biomarker of early pathology. Fluids and barriers of the CNS. 2024;21(1):97.
2. Perera C, Tolomeo D, Baker RR, Ohene Y, Korsak A, Lythgoe MF, et al. Investigating changes in blood-cerebrospinal fluid barrier function in a rat model of chronic hypertension using non-invasive magnetic resonance imaging. Frontiers in molecular neuroscience. 2022;15(NA):964632–NA.
3. Lee H, Ozturk B, Stringer MS, Koundal S, MacIntosh BJ, Rothman D, et al. Choroid plexus tissue perfusion and blood to CSF barrier function in rats measured with continuous arterial spin labeling. Neuroimage. 2022;261:119512.
4. Petitclerc L, Hirschler L, Wells JA, Thomas DL, van Walderveen MAA, van Buchem MA, et al. Ultra-long-TE arterial spin labeling reveals rapid and brain-wide blood-to-CSF water transport in humans. Neuroimage. 2021;245:118755.
5. Zhang Y, Xu K, Liu Y, Erokwu BO, Zhao P, Flask CA, et al. Increased cerebral vascularization and decreased water exchange across the blood-brain barrier in aquaporin-4 knockout mice. PLoS ONE [Electronic Resource]. 2019;14(6):e0218415.
6. Huber VJ, Igarashi H, Ueki S, Kwee IL, Nakada T. Aquaporin-4 facilitator TGN-073 promotes interstitial fluid circulation within the blood-brain barrier: [17O]H2O JJVCPE MRI study. Neuroreport. 2018;29(9):697–703.
7. Igarashi H, Tsujita M, Kwee IL, Nakada T. Water influx into cerebrospinal fluid is primarily controlled by aquaporin-4, not by aquaporin-1: 17O JJVCPE MRI study in knockout mice. Neuroreport. 2014;25(1):39–43.
8. Igarashi H, Suzuki Y, Kwee IL, Nakada T. Water influx into cerebrospinal fluid is significantly reduced in senile plaque bearing transgenic mice, supporting beta-amyloid clearance hypothesis of Alzheimer's disease. Neurol Res. 2014;36(12):1094–8.
9. Bulat M, Lupret V, Oreskovic D, Klarica M. Transventricular and transpial absorption of cerebrospinal fluid into cerebral microvessels. Coll Antropol. 2008;32:43–50.
10. Rosenberg GA, Kyner WT, Fenstermacher JD, Patlak CS. Effect of vasopressin on ependymal and capillary permeability to tritiated water in cat. American Journal of Physiology - Renal Fluid and Electrolyte Physiology. 1986;251(3(20/3):F485–F9.
11. Levin E, Sepúlveda FV, Yudilevich DL. Pial vessels transport of substances from cerebrospinal fluid to blood. Nature. 1974;249(5454):266–8.
12. Paulson GW, Kapp JP. Movement of Sodium-22, Radioiodinated Protein, and Tritiated Water from the Cisterna Magna into the Cerebrovascular Circulation. J Neurosurg. 1967;27(2):138–41.
13. Migliore A, Paoletti, P., Villani, R. Studies on the passage of water, electrolytes and proteins into the cerebrospinal fluid in the human. Acta Neurochir (Wien). 1964;12(1):1–10.
14. Heisey SR, Pappenheimer JR, Held D. Bulk flow and diffusion in cerebrospinal fluid system of goat. Am J Physiol. 1962;203(5):775–+.
15. Coppen AJ. Abnormality of the blood-cerebrospinal fluid barrier of patients suffering from a depressive illness. Journal of neurology, neurosurgery, and psychiatry. 1960;23(2):156–61.
16. Bering EA, Jr. Studies on the role of the choroid plexus in tracer exchanges between blood and cerebrospinal fluid. J Neurosurg. 1955;12(4):385–92.
17. Bering EA, Jr. Water exchange in the brain and cerebrospinal fluid; studies on the intraventricular instillation of deuterium (heavy water). J Neurosurg. 1954;11(3):234–42.
18. Bering EA, Jr. Water exchange of central nervous system and cerebrospinal fluid. J Neurosurg. 1952;9(3):275–87.

Supplementary Table 3. Magnitude estimates of water movement between blood and CSF

Reference	Model	Compartment	Values	Interpretation
16, 18	Dog, human	Ventricular & cisternal CSF	Half-times: 2–8 min (cisternal) to 8–37 min (ventricular, lumbar)	Rapid equilibration → several CSF volumes exchanged per hour
14	Goat	Ventricular CSF	Permeability coefficient 1.7 cm³/min for T_2O vs. 0.18 for Na^+	Suggests water efflux ≈ 1–2 mL/min through ventricular wall in goat brain (~2–3 % of CSF volume per sec)
12	Monkey	Cisterna magna CSF→blood	T_2O equilibrated in 10 min	Entire CSF volume exchanges with blood several times per hour
11	Dog	Pial vessels	Half-time ~14 s; complete loss in <1 min	Extremely fast microvascular clearance near pia (~100× faster than ventricular CP flow)
13	Human	Ventricular & lumbar CSF	Blood–CSF equilibrium in 1.5 h	Roughly 10–15 % of total CSF volume exchanged per 10 min
3	Rat	BCSFB water flow at CP	65–76 mL/min/100 g (~25 % of CP perfusion)	Suggests active water delivery to CSF on the order of 0.1–0.2 mL/min per rat brain (~1 mL/min scaled to human CP mass).
2	Rat	BCSFB-mediated water delivery	9–14 µL/min (ventricular CSF)	Indicates measurable flow comparable to classical CSF secretion rate.
4	Human	Blood→CSF transfer constant	Mean ~60 s turnover time (CP and SAS); CSF-ASL signal widely distributed	Implies that ~1 CSF volume could equilibrate with blood every 1–2 h → on the order of 1–2 mL/min blood → CSF exchange rate across entire craniospinal system.
**Interpretation:** Exchange half-times of 2–37 min imply blood ↔ CSF turnover rates of roughly 3–10 CSF volumes per day (total CSF volume ~150 mL), corresponding water blood-CSF water exchange at rest about 0.5–2 mL/min. In comparison, traditional figures for CSF production rates in choroid plexus are ~0.3–0.4 mL/min. These findings therefore indicate that diffusive blood ↔ CSF water exchange can match or exceed choroid-plexus-driven flow, reinforcing that CSF water homeostasis cannot be ascribed to secretion alone. There is faster exchange in cisternal and spinal subarachnoid spaces ($t_{1/2}$ ~ 3–7 min) than in ventricular CSF ($t_{1/2}$ ~ 8–37 min), indicating that both cranial (ventricular, cisternal, cortical) and spinal surfaces contribute substantially to total CSF–blood water turnover.				
